# 宣威肺癌患者肺组织中无机物的赋存与NF-κB信号通路激活以及iNOS表达的相关研究

**DOI:** 10.3779/j.issn.1009-3419.2016.01.04

**Published:** 2016-01-20

**Authors:** 加鹏 杨, 光剑 李, 云超 黄, 联华 叶, 永春 周, 光强 赵, 玉洁 雷, 小波 陈, 昆 王, 颖 陈, 春 戴, 艳军 张

**Affiliations:** 650118 昆明，昆明医科大学第三附属医院/云南省肿瘤医院胸外一科，云南省肺癌重点实验室 Department of Toracic Surgery 1 Ward, Te Tird Afliated Hospital of Kunming Medical University/Yunnan Provincial Tumor Hospital/Yunnan Key Laboratory of Lung Cancer, Kunming 650118, China

**Keywords:** 宣威, 肺肿瘤, 信号通路, Xuanwei, Lung neoplasms, Signaling pathways

## Abstract

**背景与目的:**

室内空气污染不仅会诱发哮喘，也会导致慢性阻塞性肺疾病(chronic obstructive pulmonary disease, COPD)，甚至促进肺癌发生。随着宣威肺癌的病因学研究，发现室内空气污染最终造成肺部无机颗粒物的沉积，这些物质可以造成肺泡细胞损伤、信号通路激活，最终促进肿瘤的发生。本研究旨在探讨宣威肺癌患者肺部中无机杂质的赋存以及核转录因子(nuclear factor κB, NF-κB) -诱导型一氧化氮合成酶(inducible nitric oxide synthase, iNOS)信号通路的激活情况。

**方法:**

选取48例2013年12月-2014年11月在昆明医科大学第三附属医院行手术治疗的宣威肺癌患者与其他地区的肺癌患者作为研究对象，用透射电镜(transmission electron microscope, TME)对患者术后标本进行超微结构的观察，探究无机颗粒物的赋存情况；对患者的血清行细胞因子检测；对术后的标本行免疫组化以及蛋白质印迹(Western blot)，了解NF-κB-p65蛋白以及iNOS蛋白的表达；对肺癌组织中和尿液中的8-OHdG赋存进行检测。

**结果:**

在宣威肺癌患者癌旁组织的肺泡Ⅱ型细胞、巨噬细胞中可见到大量纳米级无机物赋存；对无机物进行元素分析，含有硅(Silicon, Si)成分；宣威地区患者血清中白介素(interleukin, IL) -1β(31.50±19.16) pg/mL较其他地区肺癌患者(11.33±6.94) pg/mL高，差异有统计学意义(*P* < 0.01)；宣威肺癌与其他地区肺癌患者的术后病理组织中癌组织有NF-κB-p65和iNOS表达，较非宣威地区明显升高；癌旁和正常组织之间未见明显差异；宣威肺癌组织和尿液8-OHdG较非宣威地区肺癌患者高，肺癌患者尿液中的8-OhdG(40.124±8.597) ng/mgCr与其他地区患者(25.673±7.986) ng/mgCr相比，差异有统计学意义(*P* < 0.05)。

**结论:**

肺部无机物的赋存以及NF-κB-iNOS信号通路的激活可能促进了宣威肺癌的发生。

## Abstract (en)

**Background and objective:**

Indoor air pollution induces asthma, leads to chronic obstructive pulmonary disease, and may promote lung cancer. Our previous studies found that the accumulation of inorganic particulate mater that is due to indoor air pollution can lead to damage to alveolar cells and activation of signaling pathway, and ultimately provoke tumorigenesis. Te aim of this study is to explore the accumulation of inorganics and activation of nuclear factor κB (NF-κB)-inducible nitric oxide synthase (iNOS) signaling pathway of lung tissue in Xuanwei lung cancer patients.

**Methods:**

From December 2013 to November 2014, 48 cases Xuanwei patients with lung cancer who underwent surgical treatment from the Tird Afliated Hospital of Kunming Medical University were enrolled in this study and compared with lung cancer patients from other regions. Te ultrastructure of postoperative specimens was observed by transmission electron microscopy (TEM) to explore the occurrence of inorganic particles. Serum cytokines were analyzed. Ten, the expression levels of NF-κB-p65 protein and iNOS protein in postoperative specimens was explored by immunohistochemistry and Western blot. Finally, 8-OHdG accumulation in lung cancer tissues and urine was measured.

**Results:**

A large number of nanoscale inorganics were observed in alveolar type Ⅱ cells and macrophages located in adjacent tissues of lung cancer with Xuanwei patients. Silicon (Si) content was found in inorganic elemental analysis. Te serum interleukin (IL)-1β levels (31.50±19.16) pg/mL of Xuanwei lung-cancer patients were remarkably higher than those from other regions (11.33±6.94) pg/mL (*P* < 0.01), with statistically signifcant difference. Te pathological tissues of Xuanwei lung-cancer patients express NF-κB-p65, and iNOS expression were signifcantly higher than those of patients from non-Xuanwei regions. No signifcant di?erence was found between cancerous and normal adjacent tissues. Xuanwei lung-cancer tissues and urine 8-OHdG level (40.124±8.597) ng/mgCr were signifcantly higher than those of patients from other regions (25.673±7.986) ng/mg Cr (*P* < 0.05), with statistically signifcant di?erence.

**Conclusion:**

Te accumulation of inorganics and the activation of NF-κB-iNOS signaling pathway may contribute to Xuanwei lung cancer.

云南宣威地区是中国肺癌的点状高发区。据2013年流行病学调查^[1]^显示，宣威地区肺癌发病率居高不下。据既往研究^[2]^表明，该地区女性的肺癌发生与使用烟煤充当生活燃料密切相关。烟煤燃烧产物中富含可吸入细颗粒物以及苯并芘等有害物质。吸入肺部的无机细颗粒常常造成氧化损伤甚至激活核转录因子(nuclear factor κB, NF-κB)通路。NF-κB在人体免疫、炎症、肿瘤的发生、发展等方面发挥着重要作用^[3]^。这些有害物质可以通过激活NF-κB信号通路，产生炎症介质共同构成肿瘤的肿瘤微环境(tumor microenvironment, TME)，诱导型一氧化氮合成酶(inducible nitric oxide synthase, iNOS)与氧化损伤共同作用，损伤DNA，对肿瘤的发生起到了促进作用。本研究通过研究宣威肺癌患者术后病理组织的超微结构以及组织中NF-κB信号激活以及下游产物表达情况，进而探讨该地区肺癌的发病机制。

## 1 材料与方法

### 1.1 材料

选取2013年12月-2014年11月在昆明医科大学第三附属医院行手术治疗的宣威地区患者3 5例，男性19例，女性16例；年龄为(57.09±8.79)岁；非宣威地区肺癌手术患者1 3例，男性8例，女性5例；年龄(52.00±7.57)岁；并确诊为肺腺癌的病理标本(癌、癌旁组织、正常肺组织)(包括蜡块、冻存组织)。术前一天抽取静脉血，留取晨尿。实验试剂主要有：maxvision^TM^免疫组化试剂盒(福州迈新生物技术开发公司150804448F)细胞因子检测试剂盒(华美生物)，NF-κB p65(CST D14E12)，iNOS(GENE TEX 821502059)，8-OHdG抗体(SANTA CRUZ 11014)，8-OHdG ELISA检测试剂盒(江莱生物KB12002)。

### 1.2 实验仪器

紫外分光光度计(Beckman, USA)；电泳仪(EPS 301, USA)；凝胶成像系统(UVP, USA)；电子显微镜(Olympus, Japan)；普通显微镜(Laica, German)等。

### 1.3 方法

#### 1.3.1 电子显微镜检测

术后取1 mm×1 mm×1 mm肺脏组织块，立即用4%戊二醛固定，0.1 mol/L二甲砷酸缓冲液冲洗2遍，1%四氧化饿固定，再经缓冲液冲洗，逐级丙酮脱水，环氧树脂浸透，包埋，超薄切片，醋酸铀-枸橼酸铅染色。透射电镜观察肺脏线粒体超微结构的变化，透射电子显微镜观察宣威地区肺组织超微结构以及无机杂质的赋存；做能谱分析的病理切片不做铅染色和铀染色。

#### 1.3.2 细胞因子检测

抽取术前患者以及正常成年人的静脉血，完全凝血后，1, 500 rpm，离心15 min，含量用酶联免疫吸附法(enzyme-linked immunosorbent assay, ELISA)测定。

#### 1.3.3 免疫组化检测以及判断标准

大鼠抗人NF-κB p65浓缩型抗体，iNOS抗体，SP免疫组化染色按试剂盒说明书NF-κB p65工作浓度为1:200、iNOS工作浓度为1:100。所有切片均采用微波修复抗原，一抗4 ℃冰箱温盒内过夜，显色。用已知阳性片做对照，PBS代替一抗作阴性对照。免疫组化染色结果判断，NF-κB p56、iNOS免疫组化染色以细胞胞浆内出现棕黄色颗粒者为阳性，用双盲法，结合阳性细胞百分比及阳性细胞染色强弱两个方面计算NF-κB p56、iNOS免疫组化染色评分：染色结果判定参照1996年全国免疫组化技术与诊断标准化专题研讨会意见： < 25%的细胞着色，阴性(-)；25%-50%的细胞着色，弱阳性(+)；51%-70%的细胞着色，中度阳性(++)；> 70%的细胞着色，强阳性(+++)，本实验中只统计阳性与阴性结果。

#### 1.3.4 蛋白质印迹(Western blot)检测NF-κB p65和iNOS的表达

分别取对免疫组化中有NF-κB p65和iNOS阳性表达的宣威和非宣威地区各患者进行Western blot检测，精确称取备用的肺癌、癌旁组织、正常组织0.1 g经匀浆离心后取上清液，考马斯亮蓝G250测核蛋白浓度，并调整核蛋白浓度为5 μg/μL，置于-80 ℃保存备用。每泳道取20 μL提取蛋白以及内参GAPDH，用十二烷基硫酸钠(SDS)聚丙烯酰胺凝胶电泳(PAGE)分离，电转至固相支持体硝酸纤维素滤膜上，分别按程序加入NF-κB p65、iNOS一抗和二抗、发光试剂发光、显影^[4]^。

#### 1.3.5 DNA氧化损伤的检测

肺癌病理组织以及尿液中8-OHdG的赋存情况，SP免疫组化染色按试剂盒说明书8-OHdG工作浓度为(1:100)。所有切片均采用微波修复抗原，一抗4 ℃冰箱温盒内过夜，显色。用已知阳性片做对照，PBS代替一抗作阴性对照。然后取尿液5 mL，1, 500 rpm离心取上清后用ELISA法检测8-OHdG的含量；自动生化仪测尿液中的肌酐(creatinine, Cr)含量。

### 1.4 统计学方法

采用SPSS 17.0统计软件分析数据，各细胞因子、8-OHdG值比较采用*t*检验，NF-κB P65、iNOS免疫组化结果分析采用卡方检验，以*P* < 0.05为差异有统计学意义。

## 2 结果

### 2.1 肺组织的超微结构变化情况

在宣威肺癌的病理组织中，发现在肺泡Ⅱ型细胞(图 1A、图 1B)、巨噬细胞中可见到纳米级无机物被溶酶体吞噬(图 1C)，且不容易消化和清除。对无机物进行元素分析，含有硅成分(图 1D、图 1E)；其他地区的肺组织中未见明显的无机杂质沉积。

**1:**
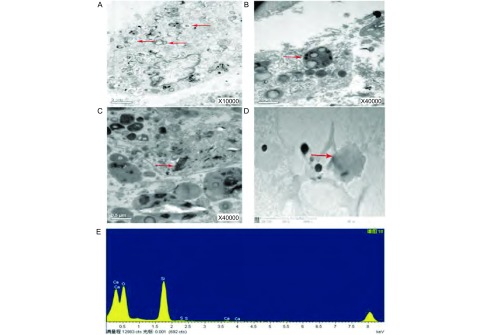
宣威肺癌患者中肺组织的超微结构与能谱图。A：肺泡Ⅱ型细胞；B：肺泡Ⅱ型细胞中的溶酶体吞噬杂质；C：巨噬细胞中的杂质；D：肺组织内的杂质；E：肺组织内的杂质行能谱分析，有硅元素。 Ultrastructure of lung tissue in Xuanwei lung cancer patients and energy spectrum. A: alveolar type Ⅱ cells; B: iveolar type Ⅱ cells lysosomal phagocytic impurities; C: macrophages impurities; D: impurities in the lung tissue; E: line spectra analysis of lung tissue, silicon element.

### 2.2 各地区的肺癌患者血清中细胞因子的表达情况

宣威地区患者血清中白介素(interleukin, IL) -1β[(31.50 ±19.16) pg/mL]较其他地区肺癌患者[(11.33±6.94) pg/mL]高，差异有统计学意义(*P* < 0.01)(表 1)。

**1:** 不同地区血清中细胞因子的表达情况（pg/mL） Cytokine expression levels in different areas (pg/mL)

Cytokines	Un-xuanwei area	Xuanwei area	*P*
IL-1*β*	11.33±6.94	31.50±19.16	< 0.01
IL-6	14.48±7.20	10.26±5.82	0.065
IL-8	24.56±14.51	35.44±17.89	0.054
TNF-*α*	40.13±16.15	47.28±22.51	0.058
IL-1*β*: interleukin-1*β*; IL-6: interleukin-6; IL-8: interleukin-8; TNF-*α*: tumor necrosis factor-*α*.			

### 2.3 各地区的肺癌患者组织中的NF-κB p65的表达情况

在宣威地区肺癌患者中，NF-κB p65蛋白主要表达与癌组织的胞核染色(图 2)，宣威肺癌患者阳性27例，阴性8例；非宣威肺癌患者阳性3例，阴性10例；差异有统计学意义(*P* < 0.05)。

**2:**
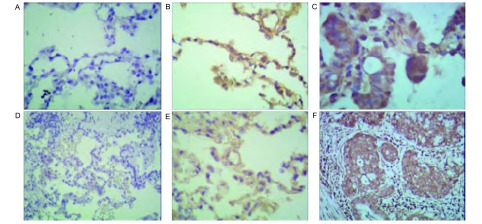
不同组织间NF-*κ*B p65表达情况(SP法)。A：宣威肺癌患者正常组织NF-*κ*B p65的表达情况(×100)；B：宣威肺癌患者癌旁组织NF-*κ*B p65的表达情况(×100)；C：宣威肺癌患者肺癌组织NF-*κ*B p65的表达情况(×200)；D：非宣威肺癌患者正常组织NF-*κ*B p65的表达情况(×100)；E：非宣威肺癌患者癌旁组织NF-*κ*B p65的表达情况(×100)；F：非宣威肺癌患者正常组织NF-*κ*B p65的表达情况(×100)。 Expression of NF-*κ*B between different organizations (SP methods). A: Xuanwei lung cancer patients with normal tissue expression of NF-*κ*B p65 (×100); B: Xuanwei lung cancer adjacent tissue expression of NF-*κ*B p65 (×100); C: Xuanwei lung cancer tissue expression of NF-*κ*B p65 (×200); D: Un-Xuanwei lung cancer patients with normal tissue expression of NF-*κ*B p65 (×100); E: Un-Xuanwei lung cancer adjacent tissue expression of NF-*κ*B p65 (×100); F: Un-Xuanwei lung cancer tissue expression of NF-*κ*B p65 (×100). NF-*κ*B: nuclear factor *κ*B.

### 2.4 各地区的肺癌患者组织中的iNOS的表达情况

在宣威地区肺癌患者中，iNOS蛋白主要表达与癌组织的胞核染色(图 3)，宣威肺癌患者阳性30例，阴性5例；非宣威肺癌患者阳性6例，阴性7例；差异有统计学意义(*P* < 0.05)。

**3:**
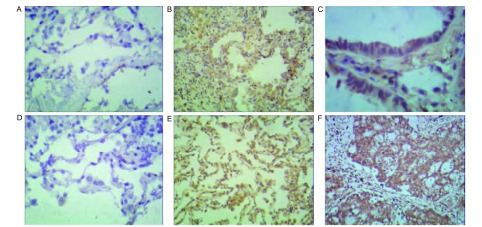
不同组织间iNOS表达情况(SP法)。A：宣威肺癌患者正常组织iNOS的表达情况(×100)；B：宣威肺癌患者癌旁组织iNOS的表达情况(×100)；C：宣威肺癌患者肺癌组织iNOS的表达情况(×200)；D：非宣威肺癌患者正常组织iNOS的表达情况(×100)；E：非宣威肺癌患者癌旁组织iNOS的表达情况(×100)；F：非宣威肺癌患者正常组织INOS的表达情况(×100)。 Expression of iNOS between different organizations (SP methods). A: Xuanwei lung cancer patients with normal tissue expression of iNOS (×100); B: Xuanwei lung cancer adjacent tissue expression of iNOS (×100); C: Xuanwei lung cancer tissue expression of iNOS; D: Un-Xuanwei lung cancer patients with normal tissue expression of iNOS (×100); E: Un-Xuanwei lung cancer adjacent tissue expression of iNOS (×100); F: UnXuanwei lung cancer tissue expression of iNOS (×100). iNOS: inducible nitric oxide synthase.

### 2.5 不同地区肺癌患者的肺组织中NF-κB p65和iNOS的表达情况

宣威肺癌与其他地区肺癌患者的术后病理组织中癌组织有NF-κB p65和iNOS表达，较非宣威地区明显升高(图 4A)；癌旁和正常组织之间未见明显差异(图 4B)。

**4:**
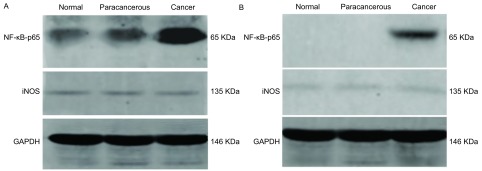
不同组织间NF-*κ*B p65和iNOS的表达情况。A：宣威肺癌各组织的NF-*κ*B p65和iNOS的Western blot结果；B：非宣威地区肺癌各组织的NF-*κ*B p65和iNOS的Western blot结果。 Expression of NF-*κ*B p65 and iNOS between different organizations. A: Expression of NF-*κ*B p65 and iNOS between different organizations in xuanwei lung cancer patients; B: Expression of NF-*κ*B p65 and iNOS between different organizations in Un-xuanwei lung cancer patients.

### 2.6 宣威肺癌患者病理组织和尿液中8-OHdG的赋存情况

在宣威肺癌组织中检测到8-OHdG以胞核染色为主，多为强阳性(图 5)，宣威肺癌患者尿液中的8-OhdG (40.124±8.597) ng/mgCr与其他地区患者(25.673±7.986) ng/mgCr相比，差异有统计学意义(*P* < 0.05) (图 6)。

**5:**
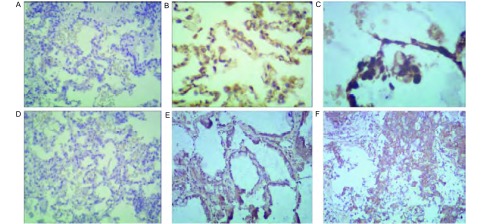
不同组织间8-OHdG表达情况(SP法)。A：宣威肺癌患者正常组织8-OHdG的表达情况(×100)；B：宣威肺癌患者癌旁组织8-OHdG的表达情况(×100)；C：宣威肺癌患者肺癌组织8-OHdG的表达情况(×200)；D：非宣威肺癌患者正常组织8-OHdG的表达情况(×100)；E：非宣威肺癌患者癌旁组织8-OHdG的表达情况(×100)；F：非宣威肺癌患者正常组织8-OHdG的表达情况(×100)。 Expression of 8-OHdG between different organizations (SP methods). A: Xuanwei lung cancer patients with normal tissue expression of 8-OhdG (×100); B: Xuanwei lung cancer adjacent tissue expression of 8-OhdG (×100); C: Xuanwei lung cancer patients with cancer tissue expression of 8-OhdG (×200); D: Un-Xuanwei lung cancer patients with normal tissue expression of 8-OhdG (×100); E: Un-Xuanwei lung cancer adjacent tissue expression of 8-OhdG (×100); F: Un-Xuanwei lung cancer patients with cancer tissue expression of 8-OhdG (×100).

## 3 讨论

云南宣威地区肺癌发病率居高不下，研究当地肺癌发病机制成为热点和难点。既往研究表明，当地肺癌高发，可能与使用劣质的燃煤作为生活燃料相关，燃煤在热力作用下释放出可吸入细颗粒物(fine particulate matter, PM2.5)、多环芳烃类(polyc yclic aromatic hydrocarbon, PAH)等有害物质。这些有害物中大部分为无机颗粒物，可能会被吸入肺内并沉积下来。本研究通过对当地肺癌患者的术后标本进行超微结构观察，在癌旁组织中的巨噬细胞、肺泡Ⅱ型细胞中发现无机物被溶酶体吞噬，且不容易消化和清除。对无机物进行元素分析，含有Si成分。在其他地区肺癌患者癌旁组织中，未见到类似无机物沉积。肺泡Ⅱ型细胞在肺损伤，炎症等过程中起着重要的作用，多数肺癌起源于Ⅱ型肺泡细胞^[5]^。且Ⅱ型肺泡细胞有部分干细胞的特性，在肺损伤的修复中起重要作用^[6]^。所以，Ⅱ型肺泡细胞内的杂质可能对肺癌的发生起到某种作用。此外一些物质虽然被吞噬细胞吞噬，由于自身难以被消化，所以被长期以次级溶酶体存在下去，也可能对机体产生损害作用。在氧化应激条件下，肺内沉积物可能参与NF-κB/IκB复合物的活化^[7]^。Lin等^[8]^研究表明，暴露于二氧化硅纳米颗粒人类支气管肺泡细胞毒性呈剂量依赖性，其细胞毒性与氧化应激密切相关。此外，氧化剂通过二氧化硅粒子和活化细胞导致细胞和肺损伤的产生；炎性细胞因子也可以调控TNF-α、IL-1β、TGF-β的表达增加，激活MAP激酶途径、NF-κB信号通路^[9]^。此外，二氧化硅粒子可以诱导细胞产生RNS和NO，也可诱导巨噬细胞和其他细胞的凋亡^[10]^。无机灰尘的吸入可以导致下呼吸道的慢性炎症^[11]^。而炎性细胞参与肿瘤发生和发展^[12]^。大气颗粒物(PM)已与肺癌的风险增加相关，甚至可能导致DNA氧化性损伤，从而促进肺癌的发生^[13]^。Dagher研究^[14]^结果表明，在体外短期暴露于PM2.5可诱导L132细胞凋亡。无机颗粒一方面通过氧化损伤等作用造成细胞直接损伤；另一方面造成一些慢性炎症，与炎症细胞及因子共同构成肿瘤微环境；导致NF-κB的信号激活，从而在宣威肺癌的发病中起到了一定的促进作用。

本研究提示，宣威地区的患者中的血液中IL-1β较其他地区患者高。研究^[15]^表明：IL-1β在胃癌的发病中起着重要的作用。Zienolddiny^[16]^研究发现：*IL-1β*基因多态性与肺癌发病风险相关联。外界的刺激物、IL-1β表达与NF-κB通路之间形成正反馈调节^[17]^。当地肺癌患者血清IL-1β较高，一方面反映了该地区肺癌的发病可能受到外界的特殊刺激，另一方面，这些刺激也会激活信号通路并共同构成肿瘤微环境，对肿瘤的发生起到促进作用。此外，IL-1β可能成为环境相关肺癌的预测指标。

本研究发现：宣威肺癌患者的术后病理组织中有NF-κB p65和iNOS蛋白表达，各组织间呈差异性表达。NF-κB最常见的形式是由p50与p65亚基组成的异二聚体，静息状态下，NF-κB与抑制因子IκB单体偶联，以无活性的形式存在于细胞质中，在外界多种因素刺激下，IκB磷酸化后降解，NF-κB得以释放并移入核内与靶基因序列上特定的结合位点结合，启动或调节基因转录，诱导iNOS的过量表达^[18]^。核转录因子NF-κB能诱导iNOS的蛋白质的表达，产生氧化应激反应，形成8-OHdG^[19]^。有研究^[20]^表明在人体内微环境下，NF-κB与细胞因子相互调节促进人膀胱癌细胞生长、侵润和转移。NF-κB作为关键性的多中心调节因子，也可以导致iNOS的表达。高表达的iNOS产生高浓度的NO，NO可通过多种机制导致DNA损伤。一氧化氮的产生与心肌肌丝和DNA的氧化损伤有关^[21, 22]^。Kankaanranta研究^[23]^表明：外源性与内源性化合物产生RNS促进了细胞癌变。Zhao等^[24]^研究表明：活性氧产生和所得慢性氧化应激过程中。罗云敬等^[25]^研究表明，iNOS是通过表达NO，形成在一氧化氮(NO·)和超(O2-·)的反应，产生强氧化剂过氧亚(ONOO-)，最终导致DNA的损伤。Burney研究^[26]^表明：过氧亚(ONOO-)诱导的DNA损伤，8-OHdG可以作为该种损伤的特异性指标。Speranza研究^[27]^表明：在肺癌中iNOS催化生产NO，分子浓度升高可以转换为高活性化合物，激活NF-κB信号通路，可诱导慢性炎症，从而增加细胞转化癌症风险。NF-κB通路一方面诱导iNOS的合成；另一方面诱导慢性炎症，为DNA损伤提供了必要的条件。

本研究表明：宣威地区肺癌患者的肺癌组织以及癌旁组织中，有不同程度的氧化损伤。这是基因突变导致肿瘤的学说基础。研究表明：DNA链断裂主要是脱氧核糖遭到羟自由基攻击破坏，磷酸二酯键的断裂或碱基的破坏或脱落^[28]^。Wu等^[29]^研究表明：活性氮(RNS)攻击细胞核和线粒体内的DNA，从而产生8-OHdG，其已成为检测和研究DNA损伤的常用指标。8-OHdG是自由基诱导DNA氧化损伤的主要形式之一，并因此被广泛用于氧化应激和癌变研究中^[30]^。宣威地区肺癌组织以及尿液中的8-OHdG升高，提示该地区肺癌发病与DNA损伤相关。

综上所述，在宣威地区肺癌高发的背后，环境有害物以及NF-κB-iNOS信号通路激活可能起到了促进肺癌形成的作用。本研究从宣威肺癌高发区的肺癌标本入手，探究了当地肺癌发生的特征以及NF-κB的激活以及iNOS的表达情况。目前对肺部无机物是否造成其他损伤以激活其他信号通路目前还有待探索，对信号通路的起始、激活的位点、以及验证有待进一步研究。希望本研究为宣威当地的环境污染的控制以及肺癌的防治提供理论依据。
